# Real-time 2D–3D door detection and state classification on a low-power device

**DOI:** 10.1007/s42452-021-04588-3

**Published:** 2021-04-29

**Authors:** João Gaspar Ramôa, Vasco Lopes, Luís A. Alexandre, S. Mogo

**Affiliations:** 1 NOVA LINCS, Costa da Caparica, Portugal; 2 Universidade da Beira Interior, Rua Marquês d’ Avila e Bolama, 6201-001 Covilhã, Portugal

**Keywords:** Door detection, Door state classification, Door segmentation, Jetson nano, 2D–3D Door dataset, Real-Time

## Abstract

In this paper, we propose three methods for door state classification with the goal to improve robot navigation in indoor spaces. These methods were also developed to be used in other areas and applications since they are not limited to door detection as other related works are. Our methods work offline, in low-powered computers as the *Jetson Nano*, in real-time with the ability to differentiate between open, closed and semi-open doors. We use the 3D object classification, *PointNet*, real-time semantic segmentation algorithms such as, *FastFCN*, *FC-HarDNet*, *SegNet* and *BiSeNet*, the object detection algorithm, *DetectNet* and 2D object classification networks, *AlexNet* and *GoogleNet*. We built a 3D and RGB door dataset with images from several indoor environments using a 3D *Realsense* camera D435. This dataset is freely available online. All methods are analysed taking into account their accuracy and the speed of the algorithm in a low powered computer. We conclude that it is possible to have a door classification algorithm running in real-time on a low-power device.

## Introduction

New mobile robots with better components and software are built daily for several purposes, from smart vacuum cleaners, [1], delivery robots, [2], security robots, [3], nursing assistant systems, [4] to intelligent housekeepers, [5], that help people with difficulties in their daily tasks.

Door detection and its state classification (we consider three possible states: closed, open or semi-open) are crucial for this type of intelligent systems to safely navigate in indoor spaces. Usually, the task of these systems implies moving between rooms and dealing with doors. It is required to provide the robot with the necessary information about the door so it can safely navigate between rooms without any problem.

Door state classification is not restricted to mobile robots and robotics, it can be applied to other problems and areas like helping visually impaired people to safely move between rooms by providing information about the existing doors and their status.

In this paper, we propose three methods for door state classification, where each one uses different information: 1) only 3D information; 2) 3D and 2D (RGB) information; and 3) only 2D (RGB) information. We focus on an approach that works in low-power systems such as the single-board computers *Nvidia Jetson Nano* or the *Raspberry Pi*. Our methods work in real-time, despite running in low-power systems with weak GPU and are based in 2D and 3D object classification, 2D object detection and 2D semantic segmentation methods. We improved our previous work dataset [6], with 3D and RGB images with three different state classes: open doors, closed doors and semi-open doors. The images were captured using a 3D Realsense Camera. The developed methods were compared in terms of test accuracy and inference speed. We used a single board computer equipped with a 3D camera and powered by a power-bank. This mobile system was used for testing the speed of our methods.

The focus of this work was in the door detection and state classification algorithms, without concerning about the rest of the robot hardware. The majority of the methods, focus on door detection only, without having to classify its state, and in some cases door handle detection for robot grasping. We propose that if the door state is classified as closed, the robot must call a human for help. If it is open the robot can simply go through it and if it is semi-open, the robot can either get around it or try to open it simply by gently pushing it. The advantage of our approach is that allows easier integration in different robot structures with different dimensions by classifying the door state with different thresholds. Our method was also tested in our developed dataset which represents more real world scenarios with more difficult cases such as obscurations, blur images, varying light conditions and different door textures.

In short, the contributions of the paper are:

- We propose three different door state classification methods, where each one uses different types of information, and all are capable of working in real-time in low-power systems.
- A labelled dataset with RGB and depth images of closed, open and semi-open doors for 2D and 3D state door classification.
- A dataset for 2D door segmentation with annotated doors and door frames.
- A dataset for 2D door detection properly annotated.

The remainder of this paper is structured as follows: Section 2 does an overview of the state-of-the-art. Section 3 describes the door state classification and detection problem. Section 4 describes the proposed methods for door classification. Section 5 describes the dataset built. Section 6 describes the experiments and results of our methods. Section 7 presents the conclusions and future work.

## Related work

There are already a vast number of studies that used door detection and classification for robot navigation tasks as moving between rooms, robotic handle grasping and others. Some have used sonar sensors with visual information, [7, 8], others used only colour and shape information, [9], or just 3D shape information, [10], some have used simple feature extractors, [11, 12] and others have used more modern methods like CNN (convolutional neural networks), [13] and the use of 3D information, [14–19].

Using visual information and ultrasonic sensors to traverse doors was an approach used in [7]. The goal was to traverse an open door with a certain opening angle using a B21 mobile robot equipped with a CCD camera sensor and 24 sonar sensors. The door traverse was divided into two sub-tasks, the door identification and the door crossing. The door identification which was the sub-task of interest for this work, used a vertical *Sobel* filter applied to the grey-scaled image. If there was a column wider than 35 pixels in the filtered image it would mean that image contained a door. The sonar sensors were used when the robot approached the door at a distance of 1 meter to confirm if it was a door or not.

The use of visual information and sonar sensors was not restricted to [7]. In [8], a 2D camera is used for long-range door detection and once detected, sonars are used to perform door classification (open doors). For the door detection, *Sobel* filters are used to detect vertical stripes in the image, which are then marked by applying to the resultant image an edge closure, composed by a generalised dilation followed by a generalised erosion. The location of possible doors is based on the expected dimensions of the doors and the direction and distance from the walls to the robot. With this information, the robot guides towards the suspected door to detect if it is an open door using the sonars sensors.

A laser-based approach for door and handle identification in indoor environments is used in [10]. In this work, a mobile manipulation platform *PR2* robot with a *Hokuyo* *UTM-30* laser sensor. The core of this approach, is to obtain 3D point clouds from the laser sensor and segment the parts of interest for door detection using robust geometric estimators and intensity distribution variations in the scan. Colour information (2D image), is not used in any part of this approach algorithm since it is highly influenced by light variations. The disadvantage of this method is that it is tested in a controlled environment. This approach was developed for detecting doors and handles using the requirements imposed by ADA (American Disability Act).

In [11], an integrated solution to recognize a door and its knob in an office environment using a humanoid platform is proposed. The goal is for the humanoid to recognize a closed-door and its knob, open the same door and pass through it. To recognize a door they match the features of the input image with the features of a reference image using the STAR Detector [20] as the feature extractor and an on-line randomised tree classifier to match the feature points. If the door is in the scene, the matched feature 3D points are computed and used so that the robot walks towards the door.

The use of colour and shape information can be sufficient for identifying features to efficiently detect doors. The approach in [9] used two neural networks classifiers for recognizing specific components of the door. One was trained for detecting the top, left and the right bar of the door and the other was trained for detecting the corners of the door. A door is detected if at least 3 of these components are detected and have the proper geometric configuration.

In [13], a method is implemented for detecting doors/cabinets and its knobs for robotic grasping using a 3D Kinect camera. It uses CNN to recognize, detect and segment the ROI (region of interest) in the image. The CNN used was the *YOLO* Detection System trained with 510 images of doors and 420 of cabinets from the *ImageNet* dataset. After obtaining the ROI, the depth information from the 3D camera is used to obtain handle point clouds for robot grasping.

Like the previous approach, in [14], a Kinect sensor is used for door detecting but, this method uses only depth information. The camera sometimes produces missing points in the depth image, and the algorithm is based in the largest cluster of missing pixels in the depth image. The total number of holes indicates the status of the door, (open or semi-open). The main advantage of this method is that it works with low-resolution depth images.

There are methods developed under a 6D-space framework, like [15], that use both colour (RGB) and geometric information (XYZ) for door detection. For detecting open doors they detect rectangular point cloud data gaps in the wall planes. The detection of closed doors is based in the discontinuities in the colour domain and in the depth dimension. It also does door classification between open and closed doors. The improved version of this algorithm, [17], can even distinguish semi-open doors using the set of points next to the door to calculate the opening angle. Another improvement in [17] was in the dataset, which is larger in size, complexity and variety.

In [16], a method is proposed that uses 3D information for door detection without using a dependent training-set detection algorithm. Initially, the point cloud containing all the scene, including the door, is prepossessed using a voxel-grid filter to reduce its density and its normal vectors are calculated. A region growing algorithm based on the pre-calculated normals is used to separate the door plane from the rest of the point cloud and after that, feature extraction is used to get the edges of the door and the doorknob.

To detect doors, 3D cameras or sonar sensors are not required, a simple RGB camera can do the job as in [12], focusing on real-time, low-cost and low-power systems. This work used the *Adaboost* algorithm to combine multiple weak classifiers into a strong classifier. The weak classifiers were based in features such as detecting pairs of vertical lines, detecting the concavity between the wall and the doorframe, texture and colour and others. They built a dataset with 309 door RGB images, 100 for training their algorithm and the rest for testing.

In [18], an approach that combines the information of neural networks with efficient point cloud processing for door and handle detection is proposed. The goal was to enable a robot (Toyota Human Support Robot) to open doors autonomously, regardless the door form or kinematic model. To detect doors and door handles the YOLO algorithm, trained in a custom dataset, was used. This dataset was built by annotating images from Open Images Dataset that contained the classes “door” and “handle”. The 3D information was used to determine the door plane normal and the handle position by computing the 6-D pose in real-time, using Regions of interest (ROIs) segmentation.

An AI-enabled framework was proposed in [19] through a Human Support Robot (Toyota HSR) for COVID-19-like pandemic situations. The role of the robot was to disinfect the door handles. It used a lightweight deep-learning (CNN Object detection) technique for the classification of the door handle image space. The robot used was also equipped with a RGBD camera and it used a NVIDIA Jetson TK1 to execute the door-handle detection. The base framework used for this task was the YOLO V3 neural network, which was trained to detect and classify door handles. The 3D information is used to convert the detected bounding box of the door handle in the 3D space.

Table 1 summarises the previous approaches and related work to detect and classify doors in indoor spaces, categorising each method studied. Although most of the approaches just do door detection and not classification of their state, as we did in this work, they have the same goal, to provide the robot with the necessary information to move between rooms, and that is the reason why we included them in this paper. The first column states whether the method uses 3D information or not. The following 3 columns state the applicability of the method (closed, open or semi-open doors). The last column focus on whether the method works in real-time or not, based on the experimental results of each method. Four of the methods do not present information regarding their speed and are marked with a “-”.

**Table 1:** Related work comparison (door detection)

Method	3D	Closed doors	Open doors	Semi-open doors	Real-time
Monasterio [7]	$\times$	$\times$	$\checkmark$	$\times$	-
Stoeter [8]	$\times$	$\times$	$\checkmark$	$\times$	$\checkmark$
Cicirelli [9]	$\times$	$\checkmark$	$\times$	$\checkmark$	$\times$
Rusu [10]	$\checkmark$	$\checkmark$	$\checkmark$	$\checkmark$	$\checkmark$
Kwak [11]	$\times$	$\checkmark$	$\times$	$\times$	$\checkmark$
Chen, [12]	$\times$	$\checkmark$	$\times$	$\times$	$\checkmark$
Llopart [13]	$\checkmark$	$\checkmark$	$\checkmark$	$\checkmark$	$\checkmark$
Yuan [14]	$\checkmark$	$\times$	$\checkmark$	$\checkmark$	-
Quintana [15]	$\checkmark$	$\checkmark$	$\checkmark$	$\times$	-
Borgsen [16]	$\checkmark$	$\checkmark$	$\times$	$\times$	$\times$
Quintana [17]	$\checkmark$	$\checkmark$	$\checkmark$	$\checkmark$	-
Arduengo [18]	$\checkmark$	$\checkmark$	$\times$	$\times$	$\checkmark$
Ramalingam [19]	$\checkmark$	$\checkmark$	$\times$	$\times$	$\checkmark$
**Ours**	$\checkmark$	$\checkmark$	$\checkmark$	$\checkmark$	$\checkmark$

## Problem definition

Mobile robots nowadays are used for multiple tasks and purposes in several indoor environments as security guard robots, tour guide robots, vacuum cleaners and others. Usually, in these environments, the robot has to navigate safely between rooms and the biggest obstacles are the doors. The mobile system normally must be able to detect the door in the scene to move to another room. In more complex situations, the robot has not only to detect the door but also has to classify its state to decide its next move.

Door detection is used in situations where the door opening is stationary and in situations where the door could either be totally open or closed.

Door state classification is useful in difficult situations where the door, in addition to open and closed, can also be semi-open. We decide to work with door state classification because it can be used by the robot to solve more complex tasks.

In this work, we focus only in the door detection and state classification using computer vision algorithms and methods without concerning the after processes and the action that the robot will take according to the opening of the door. We propose that if the door state is classified as closed, the robot must call a human for help. If it is open the robot can simply go through it and if it is semi-open, the robot can either get around it or try to open it simply by gently pushing it.

**Fig. 1:**
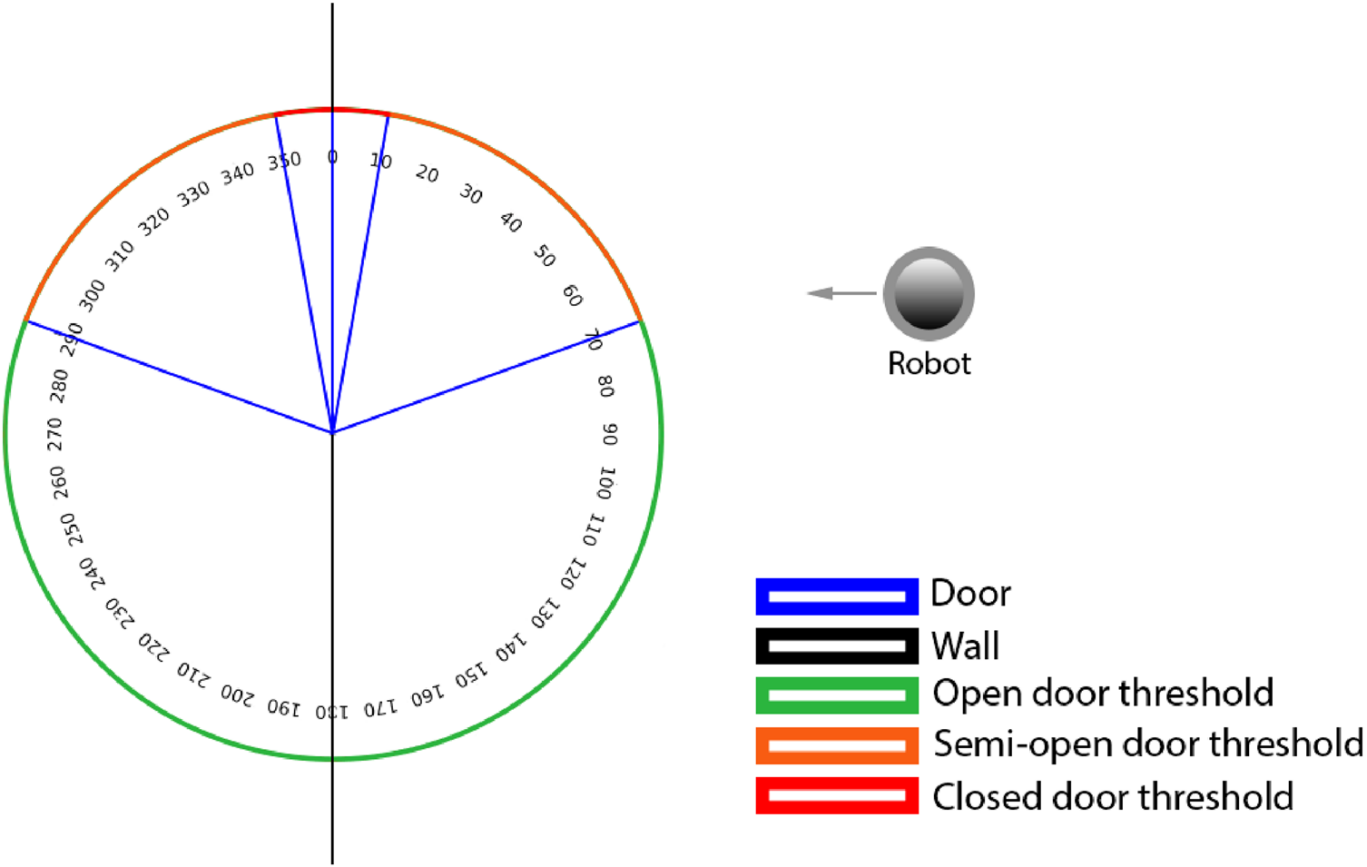
Opening angles thresholds for closed, semi-open and open doors

The door state can be classified as open, closed and semi-open depending on the door opening angle (angle between the door and the wall where the door is inserted). Doors with opening angles between 0 and 10$^{\circ }$ are closed, with opening angles between 10 and 70$^{\circ }$ are considered semi-open and with opening angles higher than 70$^{\circ }$ are considered open. We also take into account the case of doors with negative angles. This classification is done in the same way as the previous one but with the corresponding negative angles. Figure 1 treats the door opening angles thresholds from a perspective seen from above.

Although the door opening angle is the most important classification factor it is not the only one. We also have in consideration the position of the viewer in relation to the door as a classification factor. For example, the door has an opening angle of 75$^{\circ }$ but the position of the robot does not allow it to walk forward and go through the door without the need to get around it. In this case, we considered the door as semi-open, because the robot must get around it to go through it. We decide to do this approach with the objective of making this work applicable to other areas.

The main goal of the robot is to know if there is enough space to move to the next division, however, with our approach, we can further extend the applicability of this method to other problems (navigation for visually impaired people) by using the aperture angle information. The opening angle degree allows to know if a robot with different structure and dimensions such as, the *Turtlebot*, the *Savioke*, the *Cobalt* and others, can pass through the free space of the door opening and move to the other division.

It is also possible to change the opening angles thresholds for closed, semi-open and open doors based on the robot dimensions by changing the labels of the door aperture classification dataset.

## Proposed method

We propose three different methods for door state classification.

The first method, presented in Sect. 4.1, uses the combination of 2D semantic segmentation algorithms with the 3D object classification method. The second method, presented in Sect. 4.2, only uses the 3D object classification method. The third method, presented in Sect. 4.3, uses only 2D information with door detection or segmentation followed by a 2D object classification algorithm.

### Method A—2D semantic segmentation and 3D object classification

For this method, we use both RGB and depth information for door state classification, using both of our datasets.

After receiving both RGB and depth frames from the Realsense 3D camera we use a semantic segmentation method and draw a bounding box around the biggest area of pixels of the “door” class resulting from the semantic segmentation. The depth channel is aligned with the RGB channel. The depth image is cropped according to the bounding box of the RGB image, resulting in a depth image with only the door. Using the *Open3D* library [21], we converted the cropped depth image to a grey-scale point cloud. The point cloud goes to the 3D object classification *PointNet*, [22], trained with our dataset for *PointNet*, with 3 classes. The *PointNet* does the inference with the point cloud and returns the result of the classification.

**Fig. 2:**
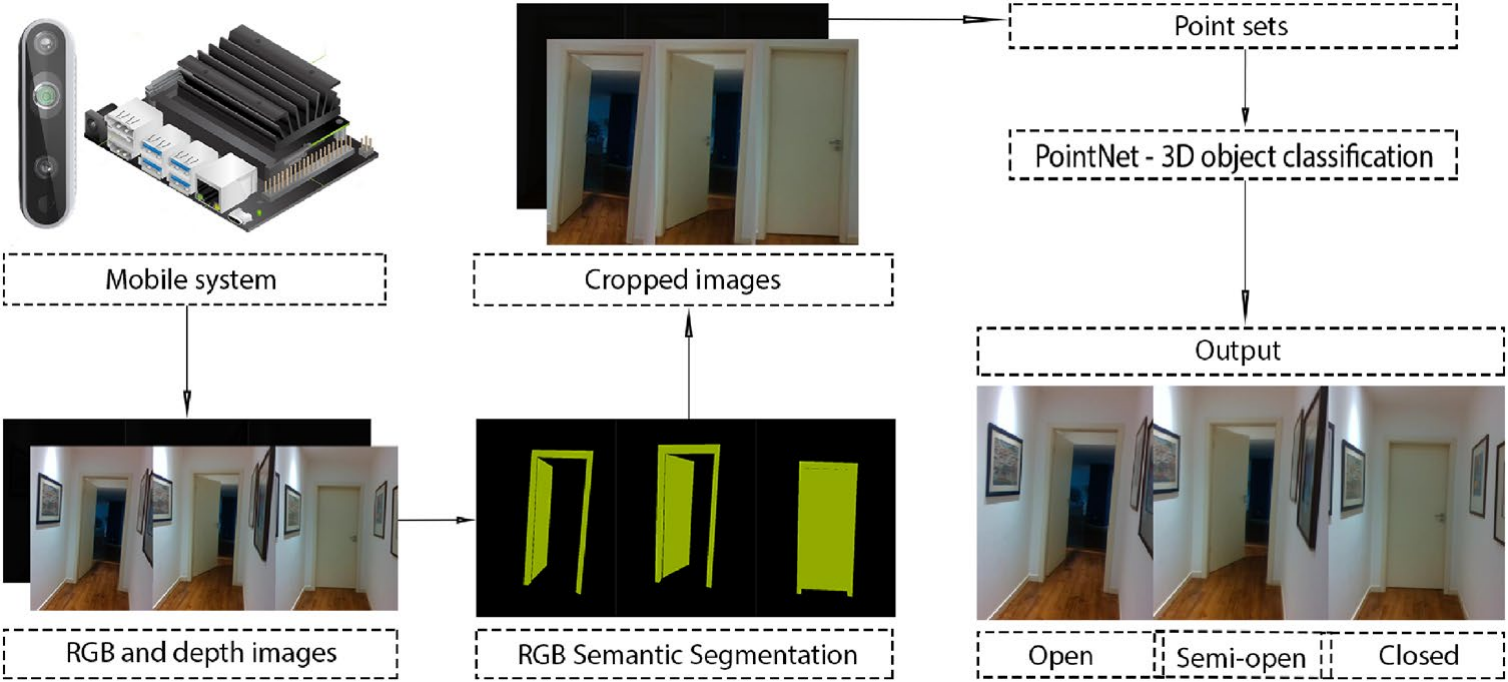
Algorithm of Method *A* (2D semantic segmentation and 3D object classification)

Figure 2 represents the described method.

Regarding the semantic segmentation algorithms we use the *FastFCN Rethinking Dilated Convolution in the Backbone for Semantic Segmentation*, [23] and the *Fully Convolutional HarDNet* which was based in the *HarDNet: A Low Memory Traffic Network*, [24]. The *FastFCN* was used because its test score was in the first three best global ranks for semantic segmentation in the *ADE20K* dataset. We also tried to implement the *EncNet*, [25] which is the network that the *FastFCN* is based on, but the implementation provided could only work in multi-GPU machines. The *ADE20K* dataset is very important for door semantic segmentation since the “door” class is labelled and it has indoor images with doors. If a semantic segmentation method performs well in this dataset it will also perform well in ours. We also used the *FC-HarDNet* because it had the best global rank metric value for real-time semantic segmentation in the *Cityscapes* dataset. We used it because it was faster than the previous method and we were pursuing a real-time door state classification method.

As the 3D object classification method, we used the *PointNet*. This method accepts unordered point sets and classifies them according to their 3D shape. We used the provided repository in [22] and changed the default dataset which was the *ShapeNet* to our dataset adjusting the data loader and the number of classes accordingly.

The difference between the methods of this family is in the 2D semantic segmentation algorithm used (*FastFCN* and *FC-HarDNet*). These methods are compared later in Sect. 6.

### Method B—3D object classification

**Fig. 3:**
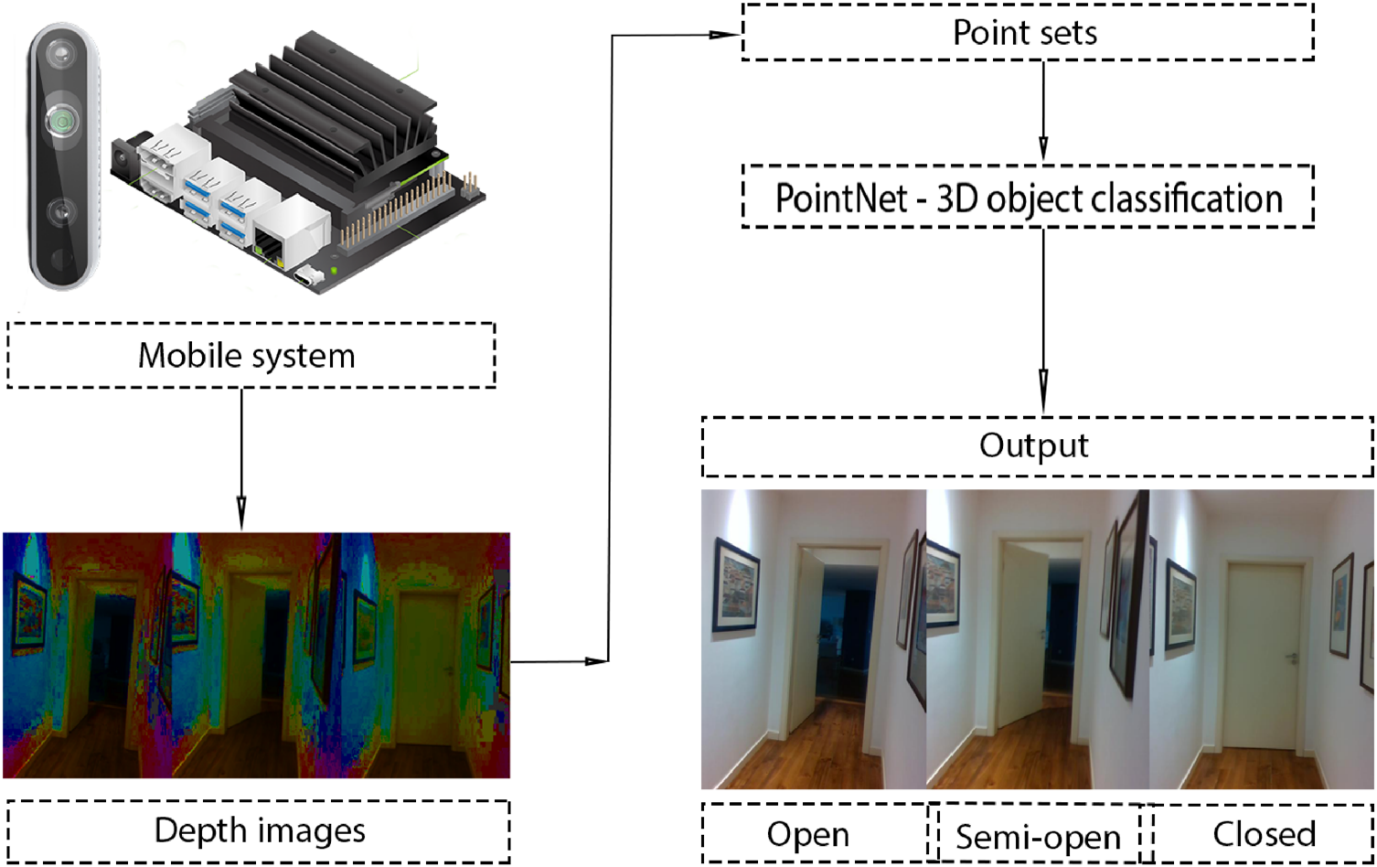
Algorithm of Method *B* (only 3D object classification)

For this method, we only used the 3D object classification method *PointNet*. Instead of receiving both RGB and depth data, we use only the depth data. The depth data is converted to a point cloud using the *Open3D* library and then converted to a point set. These point sets are the input of the *PointNet*. Unlike the previous methods, *A*, this method uses the entire point cloud without cutting it because we do not have the bounding box of the door. Although the point cloud is bigger, because it is not cropped, the number of points that enter the *PointNet* is the same. This happens because the *PointNet* has a parameter, “number of points”, which we will denote by *K*, that defines the number of points of the input point set that will be randomly selected and classified. This method’s algorithm is visually represented in Fig. 3.

This method is faster than the previous one in terms of frames per second because it does not use semantic segmentation algorithms and uses the same 3D object classification algorithm.

One parameter we can adjust in the method is if the point cloud undergoes uniform downsampling or not. The *K* parameter makes *PointNet* randomly select that number of points and if we have a big point set we might get a set that does not represent uniformly the depth data. We use the uniform downsampling algorithm from *Open3D* in the original point cloud, with approximately 300 000 points, to get a downsampled version of the same 10 times smaller. The default value of *K* was 2 500 which was too small for our point sets (300 000 or 30 000). This number was increased to 10 000 and could not be further increased because of the small GPU memory of the mobile system.

### Method C—2D door detection and 2D door state classification

For this method, we only used 2D RGB information. We tested *object detection and segmentation* algorithms because both of these types of methods can be used to obtain the door location in the input image. In Sect. 6, we further detail on the advantages and differences between using door detection and door segmentation. After obtaining the door location, the image is cropped accordingly, and we use a 2D image classification method to obtain the door state classification.

Regarding the object detection and segmentation methods we used *DetectNet* [26], *SegNet* [27], and *BiSeNet* [28]. The *DetectNet* and *SegNet* were used because they are, respectively, the neural networks primitives for object detection and semantic segmentation, provided by the *jetson-inference* repository. This repository is the Hello AI guide for deploying deep-learning inference networks into NVIDIA Jetson systems. These networks are based in *NVIDIA TensorRT* which improves inference speed and power efficiency using graph optimisations, kernel fusions, and *FP16/INT8* precision. The *BiSeNet* is a real-time semantic segmentation method and we used it since it is the fastest model with a *mIoU* precision superior to 74.7 % in the *Cityscapes dataset*. The *DetectNet* and *SegNet* were already provided in *tensorRT* format by *jetson-inference*. *BiSeNet*, on the other hand, is provided as a torch model, and we later converted it into a *TensorRT* model. To convert the model, we used the *torch onnx export method* to convert from *torch* model to a *onnx* model. After that, we used the *onnx* tool, *onnx-TensorRT tool*, to convert *onnx* to *TensorRT*. *Onnx* stands for *Open Neural Network Exchange*, and it is an open format built to represent machine learning models. We did not use the semantic segmentation algorithms of *method A* [6], since the first one: [23], was not compatible with *Jetson Nano* and the other algorithm: [24], was not capable of detecting all of the doors in the test set of the previous work dataset.

**Fig. 4:**
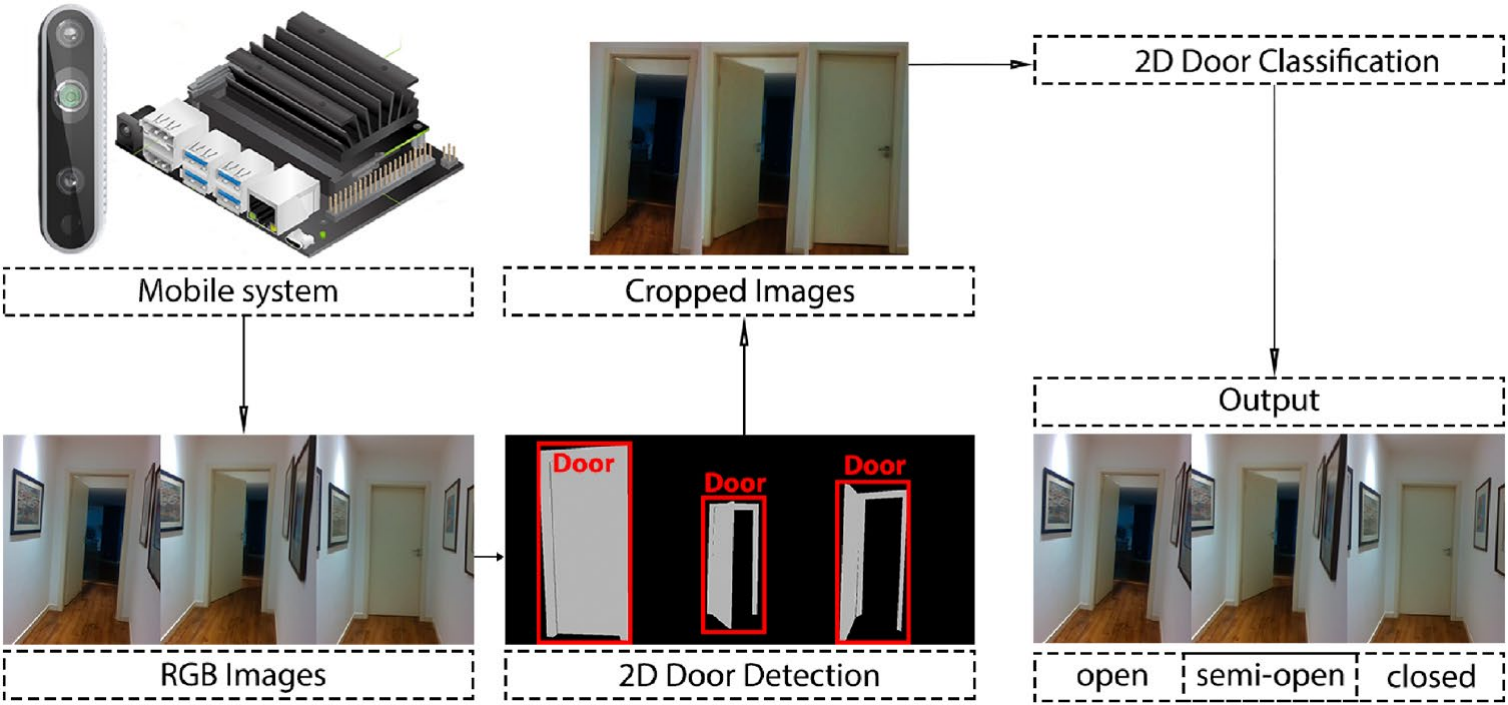
Algorithm of Method *C* (2D door detection and 2D door classification)

Regarding the object classification neural networks, we used the *AlexNet* and *GoogleNet* networks. We used these networks since they were also provided in the *jetson-inference* as the image classification primitives networks and they were already in the *TensorRT* format (Fig. 4).

One of the advantages of this method is that it does not require 3D information, thus, it does not force the system to have a 3D camera to perform door detection and state classification. Another advantage is that it provides the door detection as *method A* provides, unlike *method B*.

## Datasets

In our previous work, [6], we built two datasets, one for the 3D object classification algorithm, *PointNet* and the other for the semantic segmentation algorithms.

The first dataset is constituted by RGB images and corresponding depth images both with size of $480 \times 640$ pixels. In total, this dataset has 1206 door images, where 468 of the images are of closed doors, 588 of open doors, and 150 of semi-open doors. For the test and validation set, we used 20 samples of each class giving a total of 60 samples for test and 60 for validation. We used the remaining samples of each class to build the training set, representing a total of 1086 images.

The second dataset was built by annotating images from the previous dataset using the Computer Vision Annotation Tool (*CVAT*) [29]. This dataset is constituted by 240 grey-scaled images with the size $480 \times 640$ pixels, and it was divided into train and test sets with 200 and 40 samples respectively.

The aforementioned datasets have several disadvantages. First, the datasets are not balanced, having almost 4 times more opened door images than semi-opened door images. Second, the validation and test sets of these datasets are small, since we only have 20 samples per class on each of these sets. Finally, the semantic segmentation dataset is very small and does not have a validation set.

Due to the mentioned disadvantages, we built a new dataset that solved all of the aforementioned problems, named ***DeepDoors Version 2.0*** *Dataset*. This dataset is constituted by 3 parts, a 2D and 3D image classification part, a semantic segmentation part and an object detection part. For the first two parts, we used the previous work dataset and improve it, by collecting more data and annotating more images. The third part was built by annotating images with the *CVAT* from the image classification part.

This dataset was built using a portable system constituted by a *Raspberry Pi 3 B+* powered by a power-bank with a 3D Realsense Camera, model D435. This camera has a horizontal viewing angle (86$^{\circ }$) higher than the vertical viewing angle (57$^{\circ }$). We rotated the camera 90$^{\circ }$ to switch the angles with the purpose of including all the door area in the image. The camera was placed 135 cm above the floor.

We captured several images of doors and their surroundings with different textures and sizes. Some images have obstacles that obstruct and hide part of the door such as, chairs, tables, furniture and even persons. The goal was to create a more generic and realistic real-world dataset. We also changed the pose to get different perspectives on the same door. The images captured are from our university, public places and people’s houses. All of the images were manually captured using a portable system with a *Jetson Nano* and manually annotated using the *CVAT*, [29], for door segmentation, detection and state classification.

### Deepdoors version 2.0—door state classification

This dataset is constituted by RGB images and corresponding depth images with a size equal to $480 \times 640$ pixels. The depth images are in grey-scale with pixels values between 0 and 255 and we used a depth scale equal to 1/16. The depth in meters is equal to depth scale * pixel value, for example, if the pixel value is equal to 32 it means that that pixel is 2 m away from the viewer $(1/16 * 32 = 2)$.

**Fig. 5:**
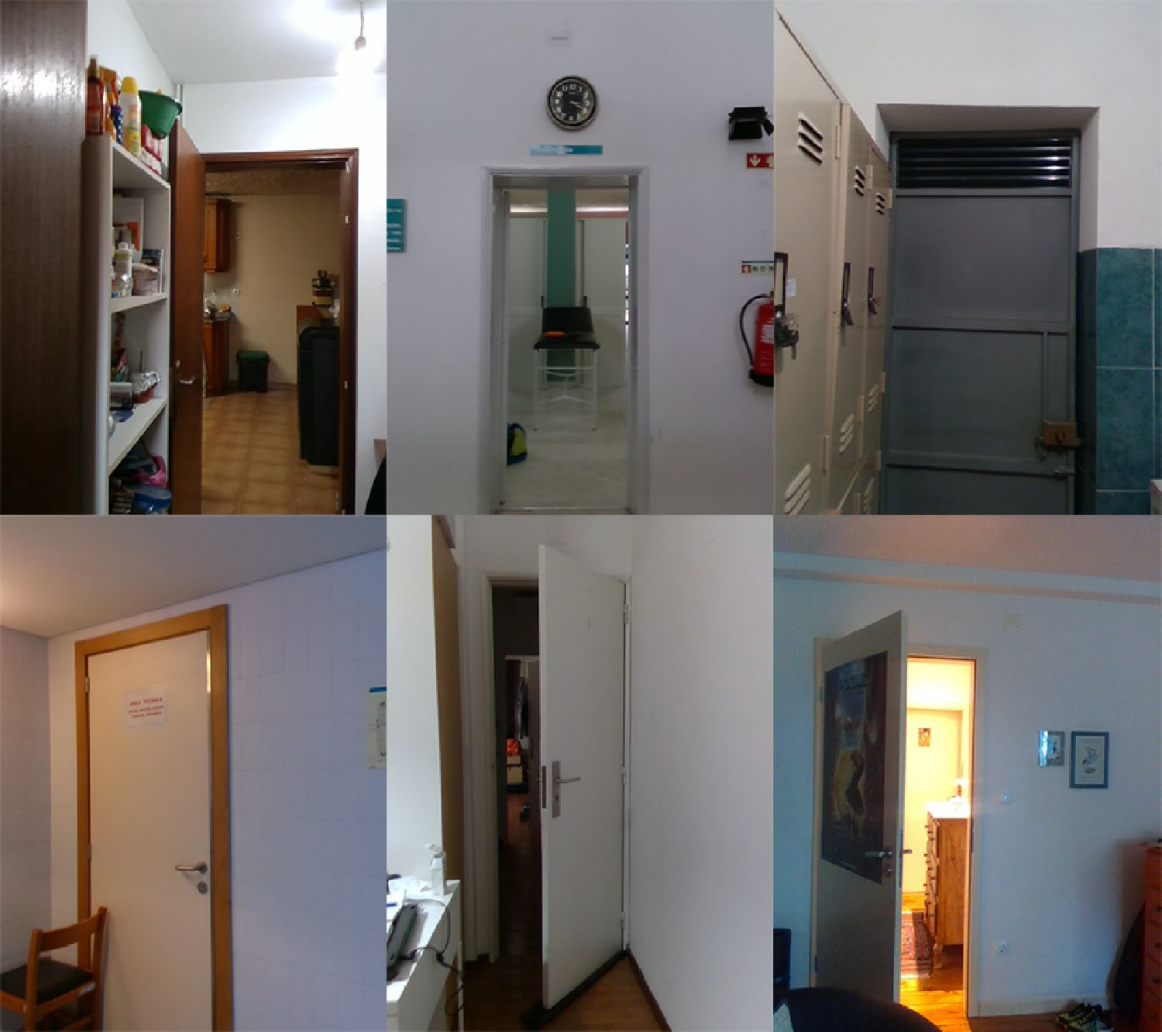
Sample images from **DeepDoors Version 2.0** dataset

In total, this dataset has 3000 door images, 1000 samples for each class: open, closed and semi-open doors. This dataset was randomly split into: 300 samples for validation, 300 for testing and 2400 for training. Figure 5, presents a few images from our dataset.

**Table 2:** Dataset comparison with previous and related work

DataSet	3D	RGB	Number of samples
Chen [12]	$\times$	$\checkmark$	309
Llopart [13]	$\times$	$\checkmark$	510
Rusu [10]	$\checkmark$	$\times$	50
Quintana [17]	$\checkmark$	$\times$	35
Arduengo [18]	$\times$	$\checkmark$	1213
Ramalingam [19]	$\times$	$\checkmark$	4500
Crivellaro [30]	$\checkmark$	$\checkmark$	3000
Previous work [6]	$\checkmark$	$\checkmark$	1206
**DeepDoors** Version 2.0	$\checkmark$	$\checkmark$	**3000**

The significance of [bold] was to highlight our number of samples in the dataset from the others datasets

We compared this dataset with our previous and the related work datasets in terms of numbers of samples and 3D/RGB coverage in Table 2. From this table, it is possible to see that our dataset has more samples than the majority of the related-work datasets and it has both RGB and depth information.

In [12, 13] and [18], the datasets built have a substantial number of samples but do not have 3D information, which incapacitates 3D-based door classification and detection methods that rely on 3D datasets. In [18], 3D information for door pose and handle detection is used. However, this method does not use a 3D dataset.

The largest dataset in terms of number of samples is the one built in [19]. However, this dataset was built by collecting images through Bing image search and using the dataset developed in [18]. It was built for door handle detection sorted into three classes, circle type, lever type and bar type handles. This dataset couldn’t be used entirely for door detection, as several images contain only the door handle zoomed with almost no information of the door itself.

The datasets developed in [10] and [17] are constituted by point clouds (3D information) but have a reduced number of samples, and the data is from a controlled environment (one only building).

We also compared our dataset with datasets developed for substantial different purposes and goals such as, the DOOR dataset built in [30]. The authors proposed a method for detecting the 3D pose of a known object using grayscale images without the need of depth sensors. The dataset has images of one non-textured door being opened and closed by a user. It contains 3000 samples that were created by using the CAD model of the door and, the ground-truth pose for all the sequences. The images were registered using the *ARUCO* marker tracking tool. Although it is used for 3D pose estimation, this dataset can be used for door state classification. The biggest disadvantage of this dataset is that, it contains only one door, consequently it is not good for model generalisation.

One advantage of our dataset, beyond the number of samples is its environment diversification. Our dataset has images from several environments and different places, such as, university facilities, house images, countryside houses with nature. Moreover, it is also constituted by several blur images to simulate a real world application, as well as obstacles that cover a substantial part of the door.

### Deepdoors version 2.0—semantic segmentation

To build a semantic segmentation dataset we used the Computer Vision Annotation Tool (*CVAT*) [29] as we did in our previous work. This dataset was built by annotating images from the image classification strand of the *DeepDoors Version 2.0* dataset. Using the polygons mode of the *CVAT*, we drew rectangles around the doors and door frames in each image.

This dataset has 3000 grey-scaled images with the size of $480 \times 640$ pixels. The splits were randomly created before annotating the images. Thus, the train set contained 2400 images, the validation set contained 300 images and the test set contained 300 images.

As we are just concerned with the doors and door frames, we only used two classes in this dataset. The pixel value is 1 if it corresponds to a door or door frame, and is 2 if it does not.

### Deepdoors version 2.0—Object Detection

This last part, was built for training and testing the object detection method *DetectNet* [26]. As for the semantic segmentation part dataset, we used the *CVAT* to annotate the images. The object detection and semantic segmentation have exactly the same number of samples, having as only difference the annotation. For annotating the images for object detection we used bounding boxes around the door and door-frame instead of using the polygon mode as we did in the semantic segmentation part. The annotations consist of the bounding boxes width minimum and maximum and height minimum and maximum.

This dataset only has 2 classes: *door*, which corresponds to a door and doorframe, and *not door*, which corresponds to all the other objects that are not doors. The splits for test, training and validation sets were exactly the same as the door segmentation strand, with the purpose to compare segmentation and detection methods trained in the same dataset and tested in the same test split.

The ***DeepDoors Version 2.0*** dataset is divided in the 3 strands, image classification (2D and 3D), semantic segmentation and object detection, and is freely available online.^1^.

## Experiments and discussion

We compared our methods against each other in real-time scenarios. We did not compare with the methods of the related work because their focus was in door detection while ours is in door state classification.

All the image classification, semantic segmentation and object detection models were trained in a machine with 16GB of RAM memory, a 256GB SDD disk, an AMD Ryzen 7 2700 processor with 16 Threads and a GeForce GTX 1080Ti graphics card with 12 GB. The mobile system where the speed tests were conducted is composed by a *Jetson Nano* in $10\,W$ mode with a Realsense 3D camera D435 with no fan.

It is important to mention that we did not change any of the algorithms used in our methods as the *PointNet*, *FastFCN*, *FC-HarDNet*, *SegNet*, *AlexNet*, *GoogleNet* and *DetectNet* with the exception of the *BiSeNet*. As it was aforementioned, the *BiSeNet* torch model was converted to a *TensorRT* model with the goal to improve the inference time. In the desktop machine, the *BiSeNet* torch model mean inference speed is 35 FPS while on the *TensorRT* model version, it was increased to 52 FPS. By converting it to a *TensorRT* model we gained 17 FPS.

For all the other algorithms we only changed the data loaders and did the necessary configurations to work with our data sets.

In our previous work, we tested the *FastFCN* and *FC-HarDNet* semantic segmentation algorithms for *Method A*, we tested one important parameter of the *PointNet* for *Method B* and then we compared both of these methods in terms of test accuracy and inference time. In this paper, we tested different computer vision algorithms that could be used in *Method C*, from 2D image classification, 2D object detection and 2D semantic segmentation algorithms. We also made a final comparison between all of the proposed 3 methods, *A, B and C*.

### Method C

*Method C* uses a 2D object detection or semantic segmentation network to detect the door to provide the necessary door information (cropped by a bounding box) to the 2D image classification network.

### Method C-Detection

For the door detection we tested one object detection network, *DetectNet* and two semantic segmentation networks, *SegNet* and *BiSeNet*.

The *DetectNet* was trained in *DIGITS (NVIDIA)* which is a GUI for training neural networks. We used our previous work dataset since we compared these results later in this paper with *Method A* and *B* and it would not be fair to compare the methods with different training and test sets. The difference between the datasets was in the annotation. Instead of 240 grey-scaled images with 2 pixel values that represent door and no-door classes, we used the *YOLO* annotation for object detection. *SegNet* and *BiSeNet* were trained with the same dataset. To compare the object detection model with the two semantic segmentation models we counted the True Positives and False Positives. We used 40 door images from the previous test set dataset and we used 40 random images with no doors from the *COCO* dataset, [31] to represent the negative cases. In *DetectNet* we used a threshold coverage value equal to 0.7. In *BiSeNet* and *SegNet* we calculated the biggest *door/doorframe* area in the semantic segmentation output using *Dilation* followed by *Erosion* filters. We used a threshold pixel area equal to 30 thousand pixels for the semantic segmentation networks.

For each method, we measured its inference time and post inference time (just for the semantic segmentation approaches) in seconds in *Jetson Nano*. The total time represents the time, in frame per second, that it takes to provide the cropped RGB image to the image classification network in Method C. Table 3 presents the evaluation and comparison of *DetecNet*, *SegNet* and *BiSeNet* on Door Detection/Segmentation in terms of the number of True Positives, False Positives, the mean inference time, post inference and total time in Jetson Nano.

**Table 3:** Evaluation and Comparison of *DetecNet*, *SegNet* and *BiSeNet* on Door Detection/Segmentation in terms of number of True Positives(TP), number of False Positives(FP), mean inference and post inference in seconds and total inference time in Jetson Nano in FPS (frames per second)

Method	TP	FP	Mean Inference(s)	Post Inference(s)	Total Inference
*DetectNet*	28/40	10/40	0.130	0	7 FPS
*SegNet*	40/40	40/40	0.400	0.006	2 FPS
*BiSeNet*	38/40	04/40	0.400	0.006	2 FPS

The *SegNet* model did not learn to segment doors, regardless of the input image, this model always returned the same output, an image with just door/doorframe pixels. That is the reason why it detected all true positive cases and got all the possible false-positive cases. Summing up, *SegNet* from the *jetson-inference* is not yet well implemented in this repository and so, it is not the most suitable door detection or segmentation network for *Method C*.

*DetectNet* works at 7 FPS in *Jetson Nano* and outputs the bounding box coordinates of the detected door, and that is why its post inference time is 0 seconds. If we compare *DetectNet* with *BiSeNet* in terms of inference speed, it is clear that *DetectNet* is faster but it is not able to detect as many doors as *BiSeNet*. In this test, *BiSeNet* only failed to segment 2 out of 40 doors and just detected 4 doors out of 40 images with no doors. *DetectNet* detected 10 fewer doors than *BiSeNet* and it detected 6 more doors in the negative images. In view of the above, we opted to use *BiSeNet* for door segmentation in *Method C*, since the results in terms of number doors detected were the best out of the 3 algorithms.

### Method C-Classification

We tested two image classification networks, the *GoogleNet* and the *AlexNet*. We used our previous work dataset for the same reasons mentioned in the previous test. We cropped the images of the dataset according to the door bounding boxes to simulate the output of the object detection or semantic segmentation method. These images were resized to $480\times 640$ since the images have different dimensions due to different crops of bounding boxes and the image classification methods required a fixed input size.

We trained both *GoogleNet* and *AlexNet* networks for 100 epochs using a learning rate equal to $2 \times 10^{-2}$ with a step-down policy and with a batch size of 32. The training was done in *DIGITS (NVIDIA)*. Initially, we tested both networks with the previous parameters and the test accuracy was $56.67\,\%$ for *AlexNet* and $36.67\,\%$ for *GoogleNet*.

The *AlexNet* model uses data augmentation by cropping the original image into a $227\times 227$ image which could lead to bad results since it just represents $16.8\,\%$ of the original image. To solve this problem we resized the images from $480\times 640$ to $227\times 227$. The other training parameters remained the same. The test accuracy was $95\,\%$. After this test, we changed the batch size from 128 to 6. As the batch size was smaller, the number of iterations per epoch increased, and for its consequent, the training time also increased, but resulted in a test accuracy of $98.33\,\%$

The *GoogleNet* model also uses data augmentation by cropping the input images but instead of $227\times 227$ images it uses $224\times 224$ images. We also resized the original training images to $224\times 224$ images and kept the other training parameters. The test accuracy was $91.67\,\%$. The default *GoogleNet* batch size is 32, and as we did for the *AlexNet*, we reduce it to 6. The highest test accuracy of all epochs after this modification the test accuracy in *GoogleNet* was $93.33\,\%$.

Table 4 represents the previous experiments in *GoogleNet* and *AlexNet*. In view of the above we conclude that the more appropriate image classification network to use in *Method C* is the *AlexNet* since it got the highest test accuracy running in real-time on low powered devices.

**Table 4:** Comparison of image classification networks for *Method C* in *DIGITS* in terms of the neural network used, training set batch size, input images size, accuracy in the test set and inference time on Jetson Nano in frames per second

Neural Network	Batch size train set	Input size	Accuracy Test	Jetson inference
*AlexNet*	128(default)	480x640	56.67	55 FPS
*GoogleNet*	32(default)	480x640	36.67	65 FPS
*AlexNet*	128(default)	227x227	95.00	55 FPS
*AlexNet*	6	227x227	**98.33**	55 FPS
*GoogleNet*	32(default)	224x224	91.67	65 FPS
*GoogleNet*	6	224x224	93.33	65 FPS

The significance of [bold] is the highest accuracy from all the neural networks used

### Method A vs Method B vs Method C

Method A and B use the 3D image classification, *PointNet* for door state classification while Method C uses the 2D image classification *AlexNet*. Method A and C use 2D semantic segmentation while Method B is more focused in real-time and just does 2D door state classification. We compared all three methods in terms of door segmentation, door state classification and total inference time in *Jetson Nano*. For door segmentation, we used the Intersection over Union as the evaluation metric in the test set and we also compared the mean inference time in seconds. For door state classification, we compared the mean accuracy in the test set and the mean inference time in seconds. All of the methods and its algorithms were trained and tested in the previous work dataset. Table 5 represents this comparison.

**Table 5:** Methods comparison in terms of door segmentation, state classification and total time in frames per second. For the semantic segmentation network, we present the mean test intersection over union and the inference time in seconds. In terms of door state classification, we show the network used, the mean test accuracy and the inference time in seconds

Method	Seg. network	Seg. IoU	Seg. time(s)	Class. network	Class. acc.	Class. time(s)	Total Time
A	*FC-HardNet*	0.418	0.131	*PointNet*	0.494	0.111	3 FPS
B	$\times$	$\times$	$\times$	*PointNet*	0.433	0.111	6 FPS
C	*BiSeNet*	0.822	0.412	*AlexNet*	0.983	0.019	1-2 FPS

Analysing the results of Table 5 we came to the conclusion that *Method C* is the most suitable for door detection and state classification in low powered devices. In terms of door segmentation *BiSeNet* from *Method C* has the highest test intersection over union, 0.822 while *FC-HardNet* from *Method A* only has 0.418. In terms of door state classification, *Method C* stands out with the highest test accuracy ($98.3\,\%$) and with the fastest inference time (0.019 s) when compared to the 3D image classification *PointNet* from *Method A and B*. The big drawback of the *Method C* is the total inference time. This method can only work at 1 to 2 FPS in *Jetson Nano*, which is slow when compared with the other methods. However, the accuracy it gains in door classification and the addition of door detection with good *IoU* test values counterbalance the processing time, making it the best methodology for door detection and state classification in this context.

### Comparing with others

We did not compare our methods with the ones from related works because those works focused on door detection, while we did door state classification. The *Llopart* method, [13], works in every class of door as it can be seen in table 1, but it does not actually do door state classification since his method cannot recognize the difference between doors, it just detects them. *Quintana*’s method, [17], is the only method that does door state classification implicitly by differentiating closed doors from open and semi-open doors using the opening angle to differentiate open from semi-open doors. Their dataset (35 point clouds) is not as complete as ours (3000 point clouds) and their method does not work in real-time, although it presented excellent results in their dataset.

## Conclusion

In this paper, we proposed three different methods for door detection and state classification to improve robot navigation and to provide it with the information to move between rooms. Each method uses different types of information, and all are capable of working in real-rime in low-power computers such as the *Jetson Nano* from *Nvidia*. The robot does not have to be connected to the internet because our methods work offline. To compare the developed methods, we used a single board computer equipped with a 3D camera powered by a power-bank. Inference speed and test accuracy was calculated and compared for each method. A novel dataset was also developed with RGB images and their respective point clouds divided into three sections, one for 2D/3D image classification with images of closed, open and semi-open doors, another for 2D semantic segmentation with annotated doors and corresponding door frames and one for 2D door detection, all properly and manually annotated.

Our work can be used in other areas and applications, as for systems that help visually impaired people navigate in indoor spaces to improve their lifestyle and other systems that use the information of the door state. Due to the fact that we used the door opening degree information, our methods can be tested in different robots with different structure and dimensions, by defining the opening angles thresholds for closed, semi-open and open doors. This work can be compared to future methods using our online published dataset and our accuracy results on *Jetson Nano*. Since our methods for door detection and state classification use publicly available algorithms, these can be tested in other datasets. Video based datasets can also be used to test our methods in future works considering that videos can be divided in a set of frames.

For future work we propose to adapt our methods to use more current *state-of-the-art* algorithms for door segmentation and door state classification. In our methods, we used *AlexNet* and *GoogleNet* networks in the *TensorRT* format for 2D door state classification. As future work, it would be interesting to extend the networks tested, considering other more powerful networks such as, *Inception V3*, [32], and *ResNet*, [33]. The same also applies for 3D door state classification and 2D door segmentation. We also desire to extend and replicate the methodologies used for object detection and classification to other scenarios and contexts within the robot navigation topic.

## Footnotes

1 https://github.com/gasparramoa/DeepDoors2
